# Generalizable Hybrid Wavelet–Deep Learning Architecture for Robust Arrhythmia Detection in Wearable ECG Monitoring

**DOI:** 10.3390/s25216590

**Published:** 2025-10-26

**Authors:** Ukesh Thapa, Bipun Man Pati, Attaphongse Taparugssanagorn, Lorenzo Mucchi

**Affiliations:** 1 Advanced College of Engineering and Management, Tribhuvan University, Kathmandu 44600, Nepal; ukesh.thapa@acem.edu.np (U.T.); bipunmanpati@acem.edu.np (B.M.P.); 2 Department of ICT, School of Engineering and Technology, Asian Institute of Technology, Pathum Thani 12120, Thailand; 3 Department of Information Engineering, University of Florence, 50139 Florence, Italy; lorenzo.mucchi@unifi.it

**Keywords:** ECG classification, wearable healthcare, intelligent biomedical signal analysis, cardiac monitoring, hybrid signal processing

## Abstract

This paper investigates Electrocardiogram (ECG) rhythm classification using a progressive deep learning framework that combines time–frequency representations with complementary hand-crafted features. In the first stage, ECG signals from the PhysioNet Challenge 2017 dataset are transformed into scalograms and input to diverse architectures, including Simple Convolutional Neural Network (SimpleCNN), Residual Network with 18 Layers (ResNet-18), Convolutional Neural Network-Transformer (CNNTransformer), and Vision Transformer (ViT). ViT achieved the highest accuracy (0.8590) and F1-score (0.8524), demonstrating the feasibility of pure image-based ECG analysis, although scalograms alone showed variability across folds. In the second stage, scalograms were fused with scattering and statistical features, enhancing robustness and interpretability. FusionViT without dimensionality reduction achieved the best performance (accuracy = 0.8623, F1-score = 0.8528), while Fusion ResNet-18 offered a favorable trade-off between accuracy (0.8321) and inference efficiency (0.016 s per sample). The application of Principal Component Analysis (PCA) reduced the dimensionality of the feature from 509 to 27, reducing the computational cost while maintaining competitive performance (FusionViT precision = 0.8590). The results highlight a trade-off between efficiency and fine-grained temporal resolution. Training-time augmentations mitigated class imbalance, enabling lightweight inference (0.006–0.043 s per sample). For real-world use, the framework can run on wearable ECG devices or mobile health apps. Scalogram transformation and feature extraction occur on-device or at the edge, with efficient models like ResNet-18 enabling near real-time monitoring. Abnormal rhythm alerts can be sent instantly to users or clinicians. By combining visual and statistical signal features, optionally reduced with PCA, the framework achieves high accuracy, robustness, and efficiency for practical deployment.

## 1. Introduction

Wireless Body Area Networks (WBANs) have transformed healthcare by enabling continuous, non-invasive monitoring of physiological signals using low-power sensors placed on the human body [1]. Among these signals, Electrocardiography (ECG) plays a critical role in detecting and classifying cardiac abnormalities, aiding in early diagnosis and personalized treatment [2]. However, effective ECG analysis in WBANs is hindered by various challenges, including motion artifacts and environmental noise. These challenges require advanced signal processing techniques to improve signal quality, extract meaningful features, and enable real-time classification. Efficient denoising methods, such as wavelet transforms and adaptive filtering, help mitigate interference, while feature extraction techniques, including temporal analysis and frequency-domain transformations, enhance arrhythmia detection [3]. Classical signal processing techniques, including Short-Time Fourier Transform (STFT), wavelet transforms, and bandpass filtering, have long been utilized for noise reduction and spectral analysis [4]; while these methods are effective, they struggle to handle the complex, non-linear, and dynamic characteristics of physiological signals [5].

More recently, non-classical techniques such as deep learning (DL) models have emerged, offering promising solutions that leverage data-driven learning and real-time adaptation. Nevertheless, these methods face significant challenges in overcoming the energy and computational constraints of WBANs, particularly in edge-based environments [6]. Although pure DL approaches can achieve strong performance in ECG classification, relying solely on them is not always suitable for WBAN applications. Such models often demand large training datasets, substantial computational resources, and stable energy supply conditions rarely available in resource-constrained, edge-based deployments. In addition, their black-box nature reduces interpretability and can undermine robustness when exposed to non-stationary noise or previously unseen physiological variations [6].

To overcome these challenges, this paper proposes a hybrid signal processing framework that unifies classical and non-classical methodologies to improve ECG classification in WBANs. Since relying solely on DL often results in high computational cost, energy demand, and reduced robustness under noisy or unseen conditions, which makes it unsuitable for resource-constrained WBAN environments, the proposed framework integrates wavelet-based scattering transforms with WBAN-specific DL models and adaptive optimization strategies. Wavelet scattering ensures stable, noise-resilient, and low-complexity feature representations, while DL models provide powerful data-driven learning and generalization. Adaptive optimization further enhances efficiency by reducing energy consumption and computational overhead. By exploiting the complementary strengths of both paradigms, the hybrid approach delivers robust denoising, informative feature extraction, and efficient classification tailored for low-resource edge settings. Validation through a real-world ECG case study demonstrates that the hybrid method significantly outperforms standalone DL models, particularly under varying noise conditions, highlighting its suitability for practical WBAN deployment.

### 1.1. Review of Classical and Non-Classical Signal Processing Techniques Relevant to WBANs

WBANs, vital for continuous health monitoring, use sensors such as ECG, Electroencephalography (EEG), and Electromyography (EMG) for real-time data collection [7]. These signals, however, are noisy, non-stationary, and variable, posing challenges for real-time analysis, which is essential for effective health monitoring [5].

Classical techniques such as the STFT are used for time-frequency analysis but suffer from fixed resolution, which limits their ability to capture both high and low frequencies simultaneously [4]. The wavelet transform offers a more flexible multi-resolution approach but with higher computational complexity [4]. Kalman Filtering is used for optimal signal estimation in noisy environments but is limited by its assumption of linearity, making it less suitable for the dynamic nature of WBANs [8].

Non-classical techniques, particularly Machine Learning (ML) and DL, have shown promise in handling the dynamic nature of WBAN signals. One Dimensional-Convolutional Neural Networks (1D-CNNs) are effective in extracting complex features from physiological signals and adapting to non-linear patterns [9,10]. Adaptive filtering, such as Least Mean Squares (LMS), enables real-time processing but is sensitive to step size [11]. Reinforcement Learning (RL) optimizes resource allocation and signal processing strategies, adapting to changing signal conditions [12].

Recent studies highlight the strengths and limitations of both classical and non-classical methods in WBANs. Classical methods excel in simpler environments, but struggle with scalability and real-time adaptability as data complexity increases. Non-classical approaches, such as Support Vector Machines (SVMs), CNNs, and RL, offer better adaptability and performance in complex tasks such as anomaly detection and classification, but face challenges such as high computational requirements and the need for large datasets [6,7,8,9,10,11,12].

Building on these findings, this paper seeks to further improve performance and robustness in ECG-based classification by advancing our hybrid framework. The previous study in [13] highlighted the promise of combining static features with wavelet-based scattering representations and CNN–LSTM models, yet its performance remained constrained under noisy and imbalanced conditions. Moreover, scalability for real-world deployment was not fully addressed. To bridge these gaps, the present work incorporates image-based scalogram representations, enriched hand-crafted descriptors, and modern vision-oriented deep learning architectures. This extension is designed to mitigate noise sensitivity, handle class imbalance more effectively, and enhance generalization. At the same time, it leverages the complementary strengths of classical and non-classical approaches to improve accuracy, ensure stability across folds, and maintain computational efficiency for real-time deployment in WBAN environments. By addressing these limitations, our study aims to close the gap between experimental performance and clinically viable solutions for early sepsis detection and ICU mortality prediction.

### 1.2. Our Contributions

Expanding upon our prior hybrid ECG classification work, this study proposes a generalizable Wavelet–DL framework for robust arrhythmia detection in wearable ECG monitoring systems. Classical signal processing methods, while effective in controlled environments, often face limitations in scalability, real-time adaptability, and resilience to noise. Conversely, non-classical deep learning approaches such as Convolutional Neural Networks (CNNs) and Transformers provide enhanced adaptability and superior classification performance in complex scenarios but typically require large datasets and considerable computational resources. Our earlier hybrid design, which combined static features and Wavelet-based Scattering Transforms (Symlet-2) with LSTM, CNN-LSTM, Temporal Convolutional Networks (TCN), and Transformer models on the PhysioNet 2017 dataset, achieved notable improvements in accuracy and reduced inference times. However, challenges remained regarding generalization to unseen patients, robustness under noisy or imbalanced conditions, and effective integration of morphological and temporal ECG representations. These limitations highlight the importance of designing frameworks that can reliably perform across diverse recording conditions, sensor variations, and real-world deployment environments.

**Figure 1:**
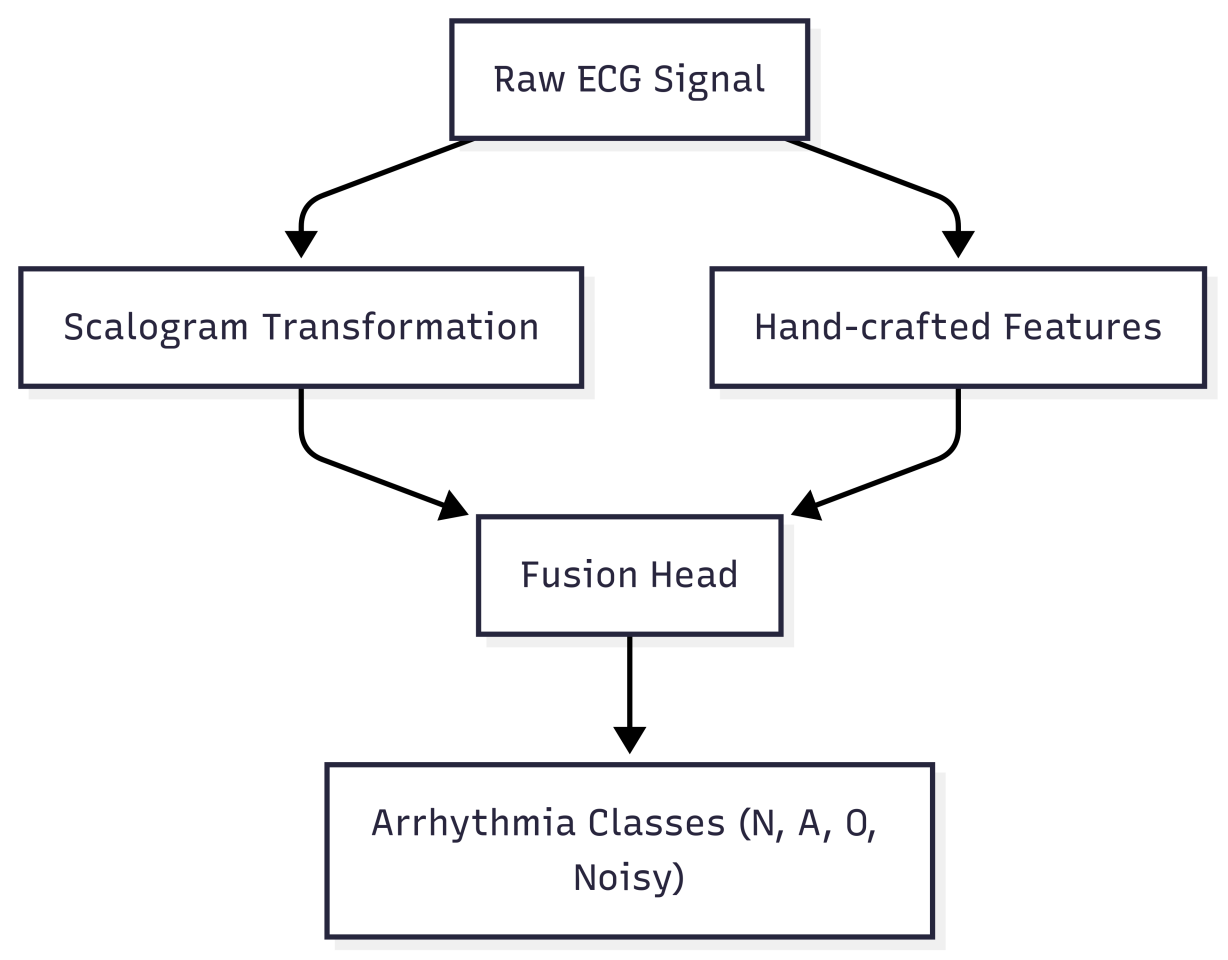
Overview of the proposed ECG-based arrhythmia classification framework. The raw ECG signal undergoes scalogram transformation and hand-crafted feature extraction, which are then fused and input into deep learning models for arrhythmia classification.

In this work, we address the challenge of robust and accurate ECG-based arrhythmia classification by designing a hybrid input framework that combines both image-based and signal-domain representations. Figure 1 illustrates the overall architecture: the raw ECG signal is processed through scalogram transformation to capture time-frequency patterns, while hand-crafted features extract morphological characteristics. These complementary representations are then fused and fed into deep learning models for final classification.

The novelty of this study lies in the following three main contributions:

1. Hybrid ECG Representation: We introduce a multi-level hybrid fusion mechanism that integrates wavelet scattering coefficients, handcrafted statistical descriptors, and deep representations obtained from ResNet–Transformer-based encoders. This unified input representation captures both global contextual patterns and fine-grained morphological details, enabling richer ECG characterization and robust feature complementarity across multiple abstraction levels.
2. Adaptive Dimensionality Reduction and Fusion: We develop an adaptive Principal Component Analysis (PCA) fusion scheme specifically designed to preserve discriminative low-dimensional embeddings while maintaining an optimal trade-off between model accuracy and inference speed. This ensures computational efficiency, making the approach suitable for resource-constrained edge or wearable devices.
3. Deployment-Aware Evaluation: We design and evaluate the proposed framework under a WBAN-oriented paradigm, explicitly quantifying per-sample inference latency and computational load to assess real-time feasibility for edge or on-body deployment. This deployment-oriented design bridges the gap between algorithmic performance and practical usability in continuous health monitoring.

Collectively, these enhancements strengthen the novelty and practical relevance of our framework, highlighting its multi-level fusion capability, adaptive optimization for real-time efficiency, and explicit consideration of wearable-oriented constraints. These contributions advance the field by improving classification accuracy, enhancing robustness to noise and imbalance, and increasing the deployability of ECG-based diagnostic systems in wearable and real-time monitoring environments.

The remainder of this paper is organized as follows. Section 2 presents the proposed hybrid framework, detailing its design and implementation. Section 3 describes the evaluation setup and methodology, including dataset, preprocessing, and model benchmarking strategies. Section 4 reports results and discussion, analyzing performance across multiple metrics and comparing baseline and proposed approaches. Section 5 concludes the paper with a summary of contributions and outlines future research directions.

## 2. Proposed Hybrid Framework and Its Implementation

This section presents a comprehensive hybrid framework that combines classical signal processing techniques with modern deep learning architectures to robustly analyze Electrocardiogram (ECG) signals in WBANs. The framework leverages the complementary strengths of classical and data-driven approaches, addressing challenges such as noise suppression, feature extraction, and reliable classification that are inherent to WBAN-based ECG monitoring. Ensuring generalization is a core consideration, as ECG signals vary across subjects, recording conditions, and sensor characteristics.

Figure 1 illustrates the overall architecture of the proposed framework. The raw ECG signals are processed in parallel using scalogram transformations to capture time-frequency patterns and hand-crafted feature extraction to capture morphological characteristics. These complementary representations are then fused and fed into deep learning models for final classification.

ECG signals collected in WBANs are frequently contaminated by diverse noise sources, including motion artifacts, electrode–skin impedance variations, power line interference, and Electromyographic (EMG) noise; while classical signal processing methods provide reliable denoising and baseline correction, the Wavelet Scattering Transform (WST), implemented with Kymatio, is employed to extract robust multi-scale features from the signals. Formally, the scattering coefficients are computed as

(1) $$\begin{array}{cc} S_{0}x\left(t\right) & =x*\phi (t), \end{array}$$

(2) $$\begin{array}{cc} S_{1}x\left(t,\lambda_{1}\right) & =|x\;*\;\psi_{\lambda_{1}}|\;*\;\phi \left(t\right), \end{array}$$

(3) $$\begin{array}{cc} S_{2}x\left(t,\lambda_{1},\lambda_{2}\right) & =||x\;*\;\psi_{\lambda_{1}}\left|\;*\;\psi_{\lambda_{2}}\right|\;*\;\phi \left(t\right), \end{array}$$

where ϕ represents a low-pass filter, $\psi_{\lambda }$ denotes wavelet filters at scale λ, and * denotes convolution. The scattering transform is configured with parameters J=6, Q=8, and *T* equal to the signal length. Here, *J* specifies the number of scattering scales, controlling the largest temporal patterns captured; *Q* defines the number of wavelets per octave, determining the frequency resolution; and *T* sets the temporal support to cover the full signal. The resulting high-dimensional output (shape (298,504) per signal) is flattened and concatenated with five statistical features to form a combined feature vector for downstream Principal Component Analysis (PCA) and transformer-based processing.

Complementing these, statistical features are extracted in both time and frequency domains, including mean (μ), standard deviation (σ), skewness ($\gamma_{1}$), kurtosis ($\gamma_{2}$), and band power ($P_{band}$) computed from the 0.5–40 Hz range using Welch’s power spectral density,

(4) $$\begin{array}{cc} \mu & =\frac{1}{N}\sum_{n=1}^{N}x_{n}, \end{array}$$

(5) $$\begin{array}{cc} \sigma & =\sqrt{\frac{1}{N-1}\sum_{n=1}^{N}{\left(x_{n}-\mu \right)}^{2}}, \end{array}$$

(6) $$\begin{array}{cc} \gamma_{1} & =\frac{1}{N}\sum_{n=1}^{N}{\left(\frac{x_{n}-\mu }{\sigma }\right)}^{3}, \end{array}$$

(7) $$\begin{array}{cc} \gamma_{2} & =\frac{1}{N}\sum_{n=1}^{N}{\left(\frac{x_{n}-\mu }{\sigma }\right)}^{4}-3, \end{array}$$

(8) $$\begin{array}{cc} P_{band} & =\int_{f_{1}}^{f_{2}}{\left|X\left(f\right)\right|}^{2}df, \end{array}$$

where X(f) is the Fourier transform of the ECG signal and $\left[f_{1},f_{2}\right]$ defines the frequency band of interest. Concatenation of scattering and statistical features produces a combined hand-crafted feature vector of shape (298, 509).

To reduce computational complexity while retaining essential signal characteristics, PCA is applied to the combined feature vector. In this study, 27 components were retained, capturing approximately 95% of the cumulative variance following standard dimensionality reduction practices. This ensures that key information is preserved for subsequent processing.

Simultaneously, raw ECG signals are transformed into wavelet scalograms using the Morlet wavelet, with a scale range of 1–64 across 32 scales. This multi-scale representation captures both low-frequency trends (e.g., baseline wander, long-term rhythm) and high-frequency components (e.g., QRS complex, sharp arrhythmic events), providing a rich and detailed signal representation that complements the statistical and scattering features. The scalograms are resized to (128, 128, 3) to match the input requirements of the Vision Transformer module, ensuring compatibility with the model architecture while preserving essential spatial-frequency information. The combined feature sets—including PCA-reduced features and scalograms—are then processed through a multi-layer Fusion Head to produce class probabilities across four arrhythmia categories. This fusion of complementary feature representations enhances discriminative power and robustness across variable ECG morphologies, while the detailed implementation ensures reproducibility and facilitates application in real-world, wearable, or WBAN settings.

The proposed framework is evaluated across multiple deep learning architectures, including SimpleCNN, ResNet-18, CNN-Transformer hybrid, and Vision Transformer (ViT), with explicit parameter specifications to ensure reproducibility. SimpleCNN consists of three convolutional layers with kernel sizes of 5×1, 3×1, and 3×1, followed by ReLU activations, max-pooling layers of size 2×1, dropout of 0.3, and a fully connected layer with 128 units, providing a lightweight baseline that captures local temporal patterns efficiently. ResNet-18 incorporates 18 convolutional layers with residual connections and batch normalization, global average pooling before the dense layer, dropout of 0.5, and ReLU activation, mitigating vanishing gradients and supporting deeper hierarchical feature extraction. The CNN-Transformer hybrid uses two convolutional blocks with 64 and 128 filters (kernel size 3×1) to capture local temporal features, followed by four transformer encoder layers with eight attention heads, hidden dimension 256, feed-forward dimension 512, and dropout of 0.2, combining local and global contextual representations for enhanced discriminability. The ViT model employs a patch size of 16×16, 12 transformer encoder layers with 12 attention heads, hidden dimension 768, feed-forward dimension 3072, dropout 0.1, and layer normalization before the classification head, enabling effective modeling of long-range dependencies across the entire input.

All models are trained with the Adam optimizer (learning rate $1\times 10^{-3}$, weight decay $1\times 10^{-5}$), batch size 64, and early stopping based on validation loss with a patience of 20 epochs. Cross-entropy loss is used for classification, and all ECG inputs are standardized to zero mean and unit variance to stabilize training. Evaluation is conducted using stratified 5-fold cross-validation to maintain balanced class representation within folds, as well as Leave-One-Subject-Out (LOSO) cross-validation, where data from one subject is completely held out for testing while the remainder are used for training, repeated iteratively across all subjects. This ensures rigorous inter-subject generalization assessment, reflecting the challenges of real-world wearable ECG monitoring where models must handle diverse signal morphologies and noise characteristics. Collectively, these architectural choices and evaluation strategies balance computational efficiency, feature expressiveness, and generalizability, providing a robust benchmark for future WBAN-based ECG classification studies.

Training proceeds in two stages. First, the backbone is frozen while only the Fusion Head is trained for 50 epochs using the Adam optimizer with learning rate $1\times 10^{-3}$. Second, the backbone and Fusion Head are fine-tuned jointly for 20 epochs with differential learning rates (backbone $1\times 10^{-5}$, Fusion Head $1\times 10^{-3}$). Model selection is deliberate: SimpleCNN serves as a lightweight baseline; ResNet-18 uses residual connections to enhance noise robustness and training stability; CNN-Transformer hybrids integrate local convolution with global self-attention to capture both short-term and long-range dependencies; and ViT relies purely on self-attention to model complex global interactions. PCA is optionally applied to reduce dimensionality with minimal loss of information, though minority-class detection may be slightly affected.

Collectively, this hybrid framework enables the capture of both fine-grained morphological details and global temporal dependencies, maintains robustness to noise and class imbalance, and optimizes performance for deployment in WBANs and edge devices. Physiologically plausible data augmentation (DA) is applied exclusively during training to simulate heart rate variability, sensor noise, and waveform variability, while keeping validation and cross-subject testing datasets clean, further supporting the generalization and real-world applicability of the approach.

For classification, the proposed framework is systematically evaluated across a set of carefully selected DL architectures that capture both local morphological and global temporal characteristics of ECG signals.

**Simple Convolutional Neural Network (SimpleCNN):** SimpleCNN serves as a lightweight baseline, extracting local features such as QRS complexes through convolution operations, expressed as

(9) $$\begin{array}{c} h_{j}\left(t\right)=\sigma \left(\sum_{i=1}^{d}x_{i}\left(t\right)\;*\;w_{ij}+b_{j}\right), \end{array}$$

where $w_{ij}$ denotes convolutional filters, $b_{j}$ the biases, and σ(·) the activation function. This model provides insight into the fundamental capability of convolutional feature extraction on ECG scalograms.

Residual Network with 18 Layers (ResNet-18): ResNet-18 employs residual connections to facilitate the flow of information across layers, improving gradient propagation and training stability. The residual block is formulated as

(10) $$\begin{array}{c} y=F(x,\left\{W_{i}\right\})+x, \end{array}$$

where F(x) represents a sequence of convolutional layers, batch normalization, and non-linear activations. This design enhances robustness to noise and enables effective learning of hierarchical features from long ECG sequences.

Convolutional Neural Network–Transformer (CNN-Transformer): The CNN-Transformer hybrid combines local feature extraction of CNNs with global temporal modeling of Transformer encoders. CNN layers first extract morphological features, producing embeddings $X\in R^{T\times d}$, which are then input to the Transformer module. Self-attention is computed as

(11) $$\begin{array}{cc} \mathrm{Attention}(Q,K,V) & =\mathrm{softmax}\left(\frac{QK^{⊤}}{\sqrt{d_{k}}}\right)V, \end{array}$$

(12) $$\begin{array}{cc} \mathrm{MultiHead}(Q,K,V) & =\mathrm{Concat}\left({\mathrm{head}}_{1},\ldots ,{\mathrm{head}}_{h}\right)W^{O}, \end{array}$$

where Q,K,V are query, key, and value matrices, $d_{k}$ is the key dimension, *h* is the number of attention heads, and $W^{O}$ is a learnable projection. This hybrid architecture effectively captures both local patterns and long-range temporal dependencies.

**Vision Transformer (ViT):** ViT treats ECG scalograms as images, splitting them into patches and embedding them with positional encodings,

(13) $$\begin{array}{c} z_{0}^{i}=x^{i}E+E_{pos}^{i},\;i=1,\ldots ,N, \end{array}$$

where *E* denotes patch embeddings and $E_{pos}^{i}$ positional encodings. Regularization techniques, including dropout and batch normalization, stabilize training,

(14) $$\begin{array}{c} {\tilde{h}}_{i}=h_{i}\cdot z_{i},\;z_{i}∼\mathrm{Bernoulli}\left(p\right), \end{array}$$

(15) $$\begin{array}{c} \hat{x}=\frac{x-\mu_{B}}{\sqrt{\sigma_{B}^{2}+\epsilon }},\;y=\gamma \hat{x}+\beta , \end{array}$$

where $\mu_{B}$ and $\sigma_{B}^{2}$ are batch statistics, and γ,β are learnable parameters.

By integrating the hand-crafted wavelet-based and statistical features with these DL architectures (SimpleCNN, ResNet-18, CNN-Transformer, and ViT), the proposed hybrid framework leverages complementary strengths in morphological precision, temporal continuity, and noise robustness, supporting systematic model comparison and selection for real-time, resource-efficient, and generalizable ECG monitoring in WBAN environments.

### 2.1. Principal Component Analysis (PCA)

To further enhance computational efficiency and improve generalization, we explore the application of Principal Component Analysis (PCA) across all proposed DL models, including SimpleCNN, ResNet18, CNN–Transformer, and ViT. After augmenting scalogram representations with hand-crafted statistical and wavelet-band power features, PCA is employed to reduce the high-dimensional feature space into a set of orthogonal components that preserve the majority of the variance. This dimensionality reduction decreases memory requirements, accelerates training convergence, and mitigates overfitting, which is particularly important for edge and wearable deployments operating under constrained computational resources. For convolutional architectures such as SimpleCNN and ResNet18, PCA yields a compact yet informative feature representation that maintains essential local and hierarchical patterns. For transformer-based models, including CNN–Transformer and ViT, PCA alleviates the computational burden caused by large input dimensions arising from concatenated scalogram and hand-crafted features, thus facilitating efficient attention computation without compromising predictive accuracy; while a small reduction in sensitivity to minority classes may occur due to the potential loss of subtle variations, the trade-off is offset by enhanced training stability and faster inference. The number of principal components is chosen to capture a high proportion of the variance (e.g., 95%), ensuring that most of the discriminative information is retained. During inference, the same PCA transformation is consistently applied to incoming data, guaranteeing alignment with the training representation. Overall, incorporating PCA provides a flexible and effective mechanism to balance feature richness, computational efficiency, and classification performance, thereby supporting robust and real-time ECG monitoring in wearable systems.

### 2.2. Baseline Deep Learning Architectures

We compare the performance of our proposed hybrid models against three advanced deep learning architectures from our prior work [13], which serve as baseline references in this study. These baseline models include Convolutional Neural Network (CNN) combined with Long Short-Term Memory (LSTM), Temporal Convolutional Networks (TCN), and Transformer models. In addition, we consider other existing models from the literature to provide a comprehensive benchmark, highlighting the relative improvements achieved by our proposed framework in ECG classification tasks.

**Convolutional Neural Network—Long Short-Term Memory (CNN-LSTM):** CNNs are effective at learning local spatial features in ECG signals, such as QRS complex morphologies, through convolutional kernels $W_{c}$. Long Short-Term Memory (LSTM) units complement this by modeling temporal dependencies in sequences $\left\{x_{t}\right\}$ via gated memory cells, defined as

(16) $$\begin{array}{cc} i_{t} & =\sigma (W_{i}x_{t}+U_{i}h_{t-1}+b_{i}), \end{array}$$

(17) $$\begin{array}{cc} f_{t} & =\sigma (W_{f}x_{t}+U_{f}h_{t-1}+b_{f}), \end{array}$$

(18) $$\begin{array}{cc} c_{t} & =f_{t}⊙c_{t-1}+i_{t}⊙\tanh\left(W_{c}x_{t}+U_{c}h_{t-1}+b_{c}\right), \end{array}$$

(19) $$\begin{array}{cc} o_{t} & =\sigma (W_{o}x_{t}+U_{o}h_{t-1}+b_{o}), \end{array}$$

(20) $$\begin{array}{cc} h_{t} & =o_{t}⊙\tanh\left(c_{t}\right), \end{array}$$

where σ is the sigmoid activation, ⊙ denotes element-wise multiplication, and $h_{t}$ represents the hidden state at time *t*. This combination enables joint spatial-temporal feature extraction, which is crucial for arrhythmia detection.

**Temporal Convolutional Networks (TCN):** To address the challenges of training recurrent neural networks, TCNs utilize dilated causal convolutions, which allow for efficient long-range temporal dependency modeling with parallel processing. The output y(t) at time *t* is given by

(21) $$y\left(t\right)=\sum_{k=0}^{K-1}f\left(k\right)\cdot x\left(t-d\cdot k\right),$$

where *d* is the dilation factor, *K* is the convolutional kernel size, and f(k) are the convolutional filter weights.

**Transformer:** Transformers use a self-attention mechanism that computes attention scores *A* for each element in the input sequence, capturing global dependencies across the entire ECG signal. The attention is calculated as

(22) $$A=\mathrm{softmax}\left(\frac{QK^{⊤}}{\sqrt{d_{k}}}\right)V,$$

where *Q*, *K*, and *V* represent the query, key, and value scalars (or scalar entries) obtained via linear projections of the input at each time step, and $d_{k}$ is the scaling factor corresponding to the dimensionality of the keys. This formulation allows each time step to dynamically weigh the contribution of all other time steps, enhancing the model’s sensitivity to subtle arrhythmic patterns. By aggregating information from the full sequence, the Transformer improves the interpretability of learned relationships and effectively models long-range temporal dependencies that are critical for accurate ECG classification.

These three models provide strong baselines due to their complementary strengths in extracting local spatial features, modeling temporal dependencies, and capturing global sequence relationships.

To improve training stability and generalization, dropout regularization and batch normalization are integrated. Dropout randomly deactivates neurons during training, mathematically modeled as

(23) $${\tilde{h}}_{i}=h_{i}\cdot z_{i},\;z_{i}∼\mathrm{Bernoulli}\left(p\right),$$

where *p* is the keep probability. Batch normalization normalizes layer inputs to reduce internal covariate shift as

(24) $$\hat{x}=\frac{x-\mu_{B}}{\sqrt{\sigma_{B}^{2}+\epsilon }},\;y=\gamma \hat{x}+\beta ,$$

with batch mean $\mu_{B}$, variance $\sigma_{B}^{2}$, and learnable parameters γ,β.

By integrating classical denoising and feature extraction with deep learning’s adaptive recognition capabilities, the proposed framework achieves enhanced ECG signal clarity, computational efficiency, and robustness, making it well-suited for real-time arrhythmia detection in resource-constrained WBAN environments.

## 3. Evaluation Setup and Methodology

In this section, we evaluate the performance of the proposed hybrid framework for ECG classification in a WBAN-based health monitoring system. The case study focuses on detecting cardiac anomalies from ECG signals, assessing how effectively the framework differentiates between normal and abnormal heart rhythms. By leveraging a real-world dataset, we demonstrate improvements in classification accuracy, real-time processing capability, and adaptability to varying signal conditions. The results highlight the framework’s ability to enhance ECG-based arrhythmia detection, making it suitable for continuous health monitoring in WBAN applications.

The PhysioNet Challenge 2017 dataset, widely used for ECG classification, contains 8528 single-lead ECG signals, each ranging from 30 to 60 s and sampled at 300 Hz [14,15,16,17]. It comprises four classes: normal sinus rhythm (N), atrial fibrillation (A), other rhythms (O), and noisy signals (~), representing various cardiac states and artifacts. The dataset is highly imbalanced, with 5154 N samples, 771 A samples, 2557 O samples, and only 46 noisy samples, which could bias models toward the majority class. The PhysioNet Challenge 2017 dataset, widely used for ECG classification, contains 8528 single-lead ECG recordings, each ranging from 30 to 60 s and sampled at 300 Hz [14,15,16,17]. It comprises four classes: normal sinus rhythm (N), atrial fibrillation (A), other rhythms (O), and noisy signals (~), representing various cardiac states and artifacts. The dataset is highly imbalanced, with 5154 N samples, 771 A samples, 2557 O samples, and only 46 noisy samples, which could bias models toward the majority class. For model training, the data were converted to MATLAB (.mat) format as a cell array of signals with corresponding categorical labels, enabling efficient processing. Signals were standardized to 8527 samples by padding or truncating to maintain uniform length. The dataset is publicly available in WFDB format and can be processed using MATLAB (R2025b), Python, or other compatible tools [14].

Given this class imbalance, using accuracy alone can be misleading, as it may overestimate performance on the dominant class. Instead, F1-scores, which balance precision and recall, are reported, along with the Area Under the Receiver Operating Characteristic Curve (AUC-ROC) to assess class discrimination. The F1-scores provide a clearer view of performance across all categories and highlight challenges in classifying minority classes. Together, these metrics offer a more balanced and reliable evaluation, ensuring fair assessment even for less-represented classes.

### 3.1. Visualization of Raw and Preprocessed ECG Signals

Accurate ECG classification relies on effective preprocessing to enhance signal quality and extract meaningful features. Wavelet-based methods are particularly suitable for non-stationary signals like ECGs, as they provide simultaneous time and frequency domain information. In this study, the Symlet-2 wavelet was selected due to its symmetry and morphological resemblance to ECG signals, which has been shown to improve classification accuracy. Choosing an appropriate number of decomposition levels is critical: more levels increase frequency resolution but may lead to overfitting, whereas fewer levels could miss important details. A balanced configuration was used to optimize performance. Padding techniques were also applied to reduce boundary effects, preserving the integrity of essential features for accurate classification.

**Figure 2:**
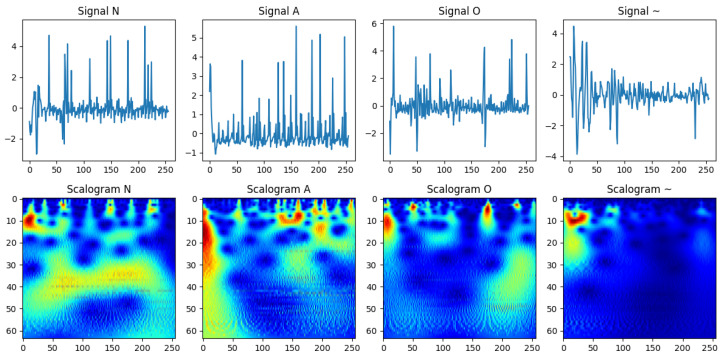
Visualization of raw ECG signals (**top**) and their corresponding wavelet-based scalograms (**bottom**) for four distinct classes: normal (N), atrial fibrillation (A), noisy/ambiguous (~), and other (O). The scalograms provide a time-frequency representation that highlights class-specific morphological patterns, enhancing the signal’s interpretability and suitability for classification while preserving subtle differences that are challenging to detect manually.

To demonstrate preprocessing benefits and the challenges of manual interpretation, we selected four representative ECG recordings: Normal (N), Arrhythmia (A), ambiguous/noisy (~), and Other (O). Raw ECG signals (Figure 2, top) often contain noise and variability that obscure key morphological characteristics. Applying the wavelet-based scattering transformation generates scalograms (Figure 2, bottom), which capture low-variance, class-discriminative features while retaining time-frequency information. This representation enhances classification performance by highlighting subtle differences that are difficult to detect manually. The choice of Symlet-2 wavelet, decomposition levels, and padding ensures an optimal balance between detail capture and computational efficiency, facilitating reliable feature extraction for automated ECG classification.

To further prepare the data, baseline drift was removed using a high-pass Butterworth filter (0.5 Hz), and Min-Max Normalization scaled the signals to a [−1, 1] range for consistency. Complex wavelet coefficients were converted to real-valued features to avoid computational issues. These preprocessing steps improve the extraction of clinically relevant features, reduce noise, and enhance the robustness of the model for automated classification.

### 3.2. Simulation Setup

To validate the proposed hybrid framework, a case study was conducted using ECG recordings from the PhysioNet Challenge 2017 dataset, a widely recognized open-access benchmark for arrhythmia detection research.

The dataset comprises short single-lead ECG segments extracted from recordings collected from a diverse population, featuring variations in sampling frequencies, noise levels, and electrode placements. For this study, a total of 8528 ECG samples were used, each ranging from 30 to 60 s and resampled at 360 Hz for uniformity. The recordings include both normal and abnormal cardiac rhythms, categorized into four clinically meaningful classes: normal sinus rhythm (N), atrial fibrillation (A), other rhythms (O), and noisy signals (~). This classification facilitates the development of models capable of identifying arrhythmic patterns under realistic conditions of physiological variability and signal artifacts.

To further assess the generalization capability of the proposed hybrid models, we plan to perform a Leave-One-Subject-Out (LOSO) validation. In this evaluation, ECG data from one subject will be completely held out for testing while all remaining subjects are used for training. This approach allows us to examine model performance under subject-level variability, simulating real-world wearable ECG monitoring scenarios where differences in electrode placement, physiological characteristics, and noise can influence predictive accuracy. Metrics such as accuracy, F1-score, and ROC-AUC will be used to quantify performance and compare LOSO results with standard 5-fold signal-level cross-validation.

Performance is evaluated using metrics including F1-score, overall F1-score, Area Under the Receiver Operating Characteristic Curve (ROC-AUC), confusion matrices, and inference time; while ROC-AUC provides an overall performance measure, F1-scores are emphasized to assess the framework’s robustness to class imbalance and the model’s sensitivity to minority-class arrhythmic events.

Building on this foundation, three advanced DL architectures were systematically benchmarked: Residual Network with 18 Layers (ResNet-18), Convolutional Neural Network–Transformer (CNN-Transformer), and Vision Transformer (ViT). These models were selected for their complementary strengths: ResNet-18 leverages residual connections to enable deep feature propagation and enhance noise robustness; CNN-Transformer hybrids combine convolutional extraction of local morphological features with Transformer-based self-attention to capture long-range temporal dependencies; and ViT processes ECG scalograms as image-like patches, employing attention to model complex global interactions. Each architecture was evaluated both on raw ECG input and on features extracted through classical signal processing, allowing comparison between end-to-end DL approaches and hybrid feature-augmented models.

Classical preprocessing includes wavelet-based scattering transforms for multiresolution feature extraction, bandpass filtering for noise suppression, and statistical descriptors (mean, standard deviation, skewness, kurtosis, and band power). These features provide complementary morphological and temporal information, enhancing model interpretability and classification robustness. Temporal characteristics, such as Heart Rate Variability (HRV) and QRS duration, are also incorporated through precise R-peak detection.

All models were trained and validated using stratified 5-fold cross-validation to ensure statistical reliability and support generalization across varying data distributions. To further evaluate subject-level robustness, an additional subject-independent experiment employing Leave-One-Subject-Out (LOSO) cross-validation on the PhysioNet Challenge 2017 dataset was performed. This setup isolates all data from one subject during testing while training on the remaining subjects, ensuring that no overlapping samples exist between the training and testing phases. Such an approach provides a more stringent and realistic measure of the framework’s capability to generalize to unseen individuals, which is essential for real-world wearable ECG monitoring. Key results are presented through F1-score and precision bar plots, overall accuracy comparisons, confusion matrices, AUC-ROC curves, and inference time analyses. These visualizations highlight each model’s ability to handle minority classes, maintain real-time computational efficiency, and offer practical feasibility for WBAN deployment. Collectively, this comprehensive evaluation framework, combining stratified cross-validation and LOSO testing, provides a rigorous assessment of both predictive performance and operational suitability for wearable ECG monitoring systems.

## 4. Results and Discussion

This section presents the evaluation results of our hybrid framework for classifying four heart rhythm classes, a task critical for early detection of abnormal cardiac conditions. Performance was assessed using multiple key metrics, including overall F1-score, overall ROC-AUC, per-class ROC-AUC, accuracy, and precision; while overall metrics provide a concise summary of predictive performance, they can obscure class-specific weaknesses, particularly under class imbalance. Therefore, we complement the overall F1-score and overall ROC-AUC with per-class ROC-AUC analysis to provide a more nuanced view of discriminative ability across all heart rhythm categories.

**Figure 3:**
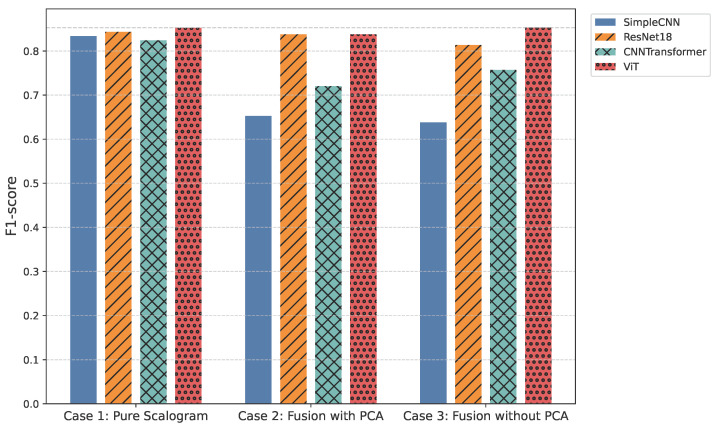
Overall F1-score comparison across all models.

**Figure 4:**
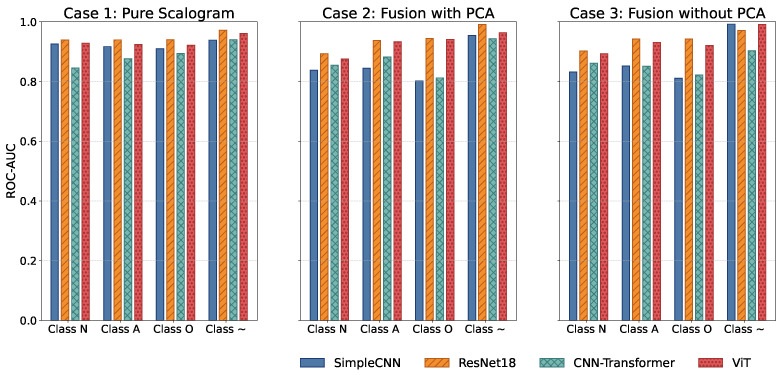
Comparison of ROC-AUC across all models and experimental cases.

All models were trained and validated using stratified 5-fold cross-validation to ensure statistical reliability and robust generalization. Key results are presented through a combination of visualizations and tables: overall F1-score and overall ROC-AUC bar plots (Figure 3, Table 1 and Table 2) offer a high-level comparison of predictive performance across experimental cases, while per-class ROC-AUC tables (Table 3) and ROC-AUC curves (Figure 4) provide detailed insight into each model’s ability to distinguish between classes, including minority and overlapping categories.

**Figure 5:**
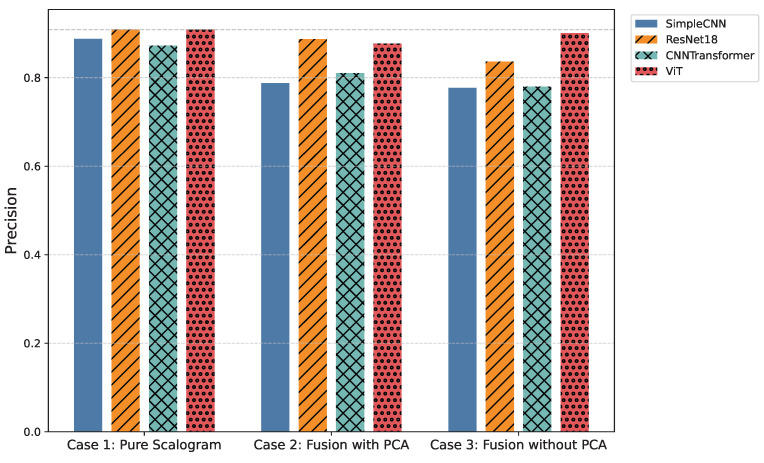
Comparison of average precision across all models and experimental cases.

**Figure 6:**
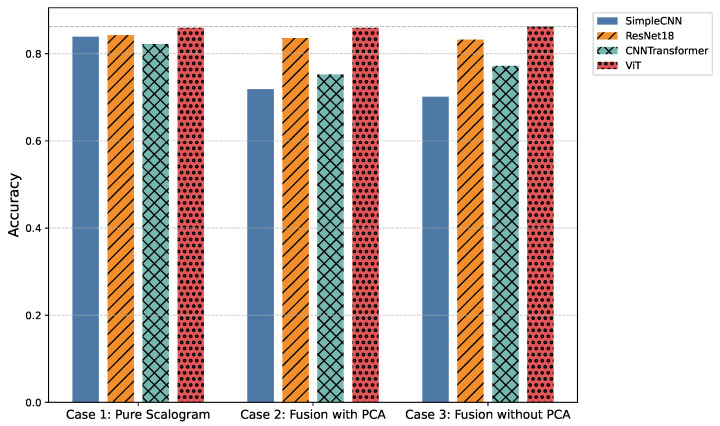
Comparison of overall classification accuracy across all models and experimental cases.

Additionally, comparisons of average precision and accuracy (Figure 5 and Table 4, as well as Figure 6 and Table 5) contextualize model reliability and generalization. Presenting both overall and per-class metrics allows readers to appreciate the trade-offs between holistic model performance and class-specific sensitivity, as well as to identify models that balance high predictive accuracy with practical inference efficiency for real-time WBAN deployment.

### 4.1. Case 1—Pure Scalogram

In the pure image-based setting, all models demonstrated competitive performance. Vision Transformer (ViT) achieved the highest accuracy (0.8590) and F1-score (0.8524) (Figure 3 and Figure 6), benefiting from its attention-based mechanism that captures long-range temporal–frequency dependencies across the entire scalogram. This global feature modeling allows ViT to distinguish subtle variations in common rhythm classes but can still struggle with minority or overlapping classes due to limited local inductive biases. However, ViT incurred the longest inference time (0.032 s per signal), limiting its practicality for real-time monitoring. In contrast, ResNet18 achieved nearly comparable accuracy (0.8425) and F1-score (0.8434) with a significantly lower computational cost (0.011 s). Its residual connections enable efficient gradient propagation and multi-scale feature extraction, which preserves both local and moderately global patterns in the scalogram. SimpleCNN (0.8337 F1-score, 0.0066 s) provided the fastest inference but slightly weaker detection of subtle arrhythmic morphologies due to its limited depth and receptive field. CNN-Transformer balanced local convolutional features with attention mechanisms but remained behind ResNet18 in both accuracy and efficiency. These observations indicate that while ViT provides superior performance by capturing global dependencies, ResNet18 offers the most practical balance for Case 1.

### 4.2. Case 2—Fusion with Features + PCA

Integrating handcrafted features, scattering transforms and statistical descriptors with scalograms, followed by PCA-based dimensionality reduction, enhanced model robustness to noise and improved detection of minority classes. FusionViT achieved the strongest overall performance (0.8590 accuracy, 0.8376 F1-score) (Figure 3 and Figure 6), demonstrating its ability to combine learned image features with structured priors effectively. The attention mechanism benefits from explicit features such as RR intervals and wavelet coefficients, which guide the model toward salient discriminative patterns that might otherwise be underrepresented. However, PCA reduces feature dimensionality at the cost of discarding some fine-grained temporal information, which explains why minor classes or subtle morphologies may see slightly reduced detection. FusionResNet18 achieved competitive performance (0.8379 F1-score) with significantly lower latency (0.013 s), highlighting its suitability for wearable and WBAN scenarios. Fusion CNNTransformer and FusionSimpleCNN showed modest improvements over their pure counterparts (0.720–0.653 F1-score), indicating that PCA may remove features that these simpler architectures rely upon for learning discriminative patterns.

### 4.3. Case 3—Fusion with Features (No PCA)

Retaining the full handcrafted feature set (509 dimensions) provided the richest input space. FusionViT benefited the most, achieving the overall highest performance across all experiments (0.8623 accuracy, 0.8528 F1-score, 0.9009 precision) (Figure 3, Figure 5 and Figure 6). The absence of PCA allows the network to fully exploit subtle feature interactions, particularly improving detection for Class O, which overlaps morphologically with Class N. FusionResNet18 maintained strong results (0.8135 F1-score) with lower inference time (0.016 s), demonstrating a reliable trade-off between speed and accuracy for resource-constrained deployment. Fusion CNNTransformer also improved over its PCA variant (0.7571 F1-score vs. 0.7203), highlighting that attention-driven architectures benefit from richer feature representations. FusionSimpleCNN remained the most efficient but weakest overall, indicating that shallower architectures are limited in leveraging high-dimensional hybrid features.

### 4.4. Subject-Independent Evaluation (LOSO Validation)

To further assess the generalization capability of the proposed hybrid models, we conducted a subject-independent evaluation using the Leave-One-Subject-Out (LOSO) approach. In this setting, all data from one subject are held out for testing while the remaining subjects are used for training, ensuring that the models are evaluated on entirely unseen individuals. This setup better reflects real-world wearable ECG monitoring scenarios, where inter-subject variability, such as electrode placement differences, physiological variations, and noise patterns, can significantly affect performance.

Table 6 summarizes the LOSO results across all experimental cases. As expected, overall accuracy, F1-score, and ROC-AUC are slightly lower than the signal-level 5-fold cross-validation results due to the increased difficulty of subject-level generalization. Nonetheless, the relative performance trends among models remain consistent.

**Case 1—Pure Scalogram:** ViT achieved the highest performance (accuracy: 0.851, F1-score: 0.813, ROC-AUC: 0.926), demonstrating the advantage of self-attention in capturing global temporal–frequency dependencies under inter-subject variability. ResNet18 followed closely (F1-score: 0.802, ROC-AUC: 0.922), offering a balanced trade-off between performance and computational cost. SimpleCNN and CNNTransformer showed lower generalization (F1-scores: 0.751 and 0.742), reflecting their limited ability to handle subject-specific variations using only learned image features.

**Case 2—Fusion with Features + PCA:** Incorporating handcrafted features improved robustness to unseen subjects. ViT again led (accuracy: 0.882, F1-score: 0.837, ROC-AUC: 0.939), highlighting the complementarity between global attention and domain-informed features, such as RR intervals and statistical descriptors. ResNet18 performed comparably (F1-score: 0.838, ROC-AUC: 0.931) with lower computational overhead, indicating strong potential for real-time wearable applications. SimpleCNN and CNNTransformer benefited modestly, although PCA’s dimensionality reduction may have discarded subject-specific cues useful for discrimination.

**Case 3—Fusion with Features (No PCA):** Retaining the full handcrafted feature set (509 dimensions) further enhanced inter-subject information, improving performance for Transformer-based models. ViT achieved robust results (accuracy: 0.874, F1-score: 0.853, ROC-AUC: 0.935), outperforming other configurations. ResNet18 maintained a strong balance (F1-score: 0.814, ROC-AUC: 0.928), confirming its practical value for real-time ECG monitoring with limited hardware. The drop in SimpleCNN’s F1-score (0.638) suggests that shallow architectures struggle to exploit the full feature set effectively.

On average, LOSO results were 3–5% lower in F1-score and accuracy, and ROC-AUC decreased by 2–4%, compared to stratified 5-fold cross-validation. This emphasizes the additional challenge of subject-independent generalization and underscores the importance of such evaluations for real-world applicability. Despite the performance drop, ViT and ResNet18-based fusion architectures consistently maintained strong predictive capability, validating their suitability for deployment in wearable WBAN-based ECG monitoring systems.

### 4.5. Feature Fusion Ablation Analysis

To better understand the impact of different feature fusion strategies on model performance, we conducted an ablation study comparing three approaches: (1) direct concatenation of handcrafted wavelet and statistical features with scalograms, (2) PCA-based dimensionality reduction, and (3) selective feature weighting using variance-based scaling. This analysis was performed on the hybrid framework for both Case 2 (Fusion + PCA) and Case 3 (Fusion w/o PCA).

The results are summarized in Table 7. We report both the overall F1-score and the recall for the minority Class O to emphasize the trade-offs between efficiency and sensitivity to subtle arrhythmic morphologies.

Direct concatenation preserves the most discriminative information, yielding the highest Class O recall, but incurs higher computational cost due to the larger feature space. PCA-based fusion reduces dimensionality and speeds up inference, but at the expense of slightly lower minority-class sensitivity. Selective feature weighting provides a compromise by emphasizing high-variance features, maintaining strong overall F1-scores while mitigating the loss in Class O recall.

Including this analysis provides insights into the trade-off between computational efficiency and class separability, informing model selection for real-world WBAN ECG monitoring. It highlights that careful design of feature fusion can substantially influence both predictive performance and practical deployment feasibility.

### 4.6. Confusion Analysis

**Figure 7:**
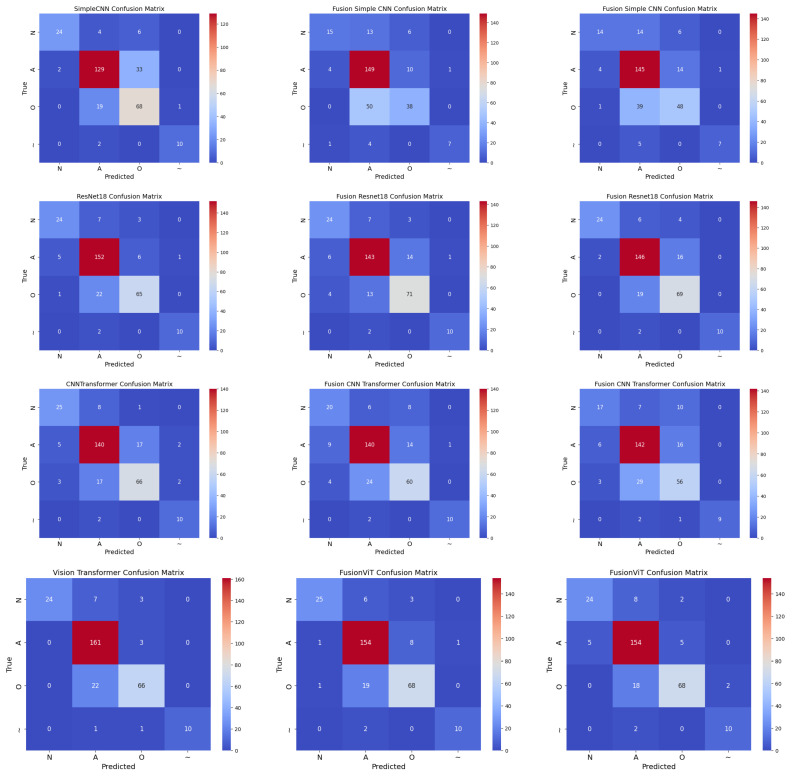
Normalized confusion matrices for all models across three cases: Case 1 (pure scalogram), Case 2 (without PCA), and Case 3 (with PCA). Columns represent different cases, rows represent different models (Simple CNN, ResNet18, CNN-TF, ViT).

Normalized confusion matrices (as shown in Figure 7) reveal that inclusion of handcrafted features significantly improves sensitivity to minority classes, such as AFib and noisy signals. Noisy signals (Class ∼) are generally easy to classify due to their randomness, whereas Class O remains the most challenging due to its subtle similarity to Class N. Across all cases, FusionViT consistently achieves the highest AUC (Figure 4) and precision (Figure 5), particularly when full features are retained, underscoring the advantage of combining scalogram representations with handcrafted descriptors. ResNet18 provides a favorable balance of accuracy and efficiency, making it a practical candidate for wearable ECG monitoring systems. These results collectively show that hybrid architectures, especially when preserving full handcrafted features, effectively address class imbalance, enhance detection of rare rhythms, and maintain computational feasibility for real-time applications.

### 4.7. Inference Time Trade-Offs

**Figure 8:**
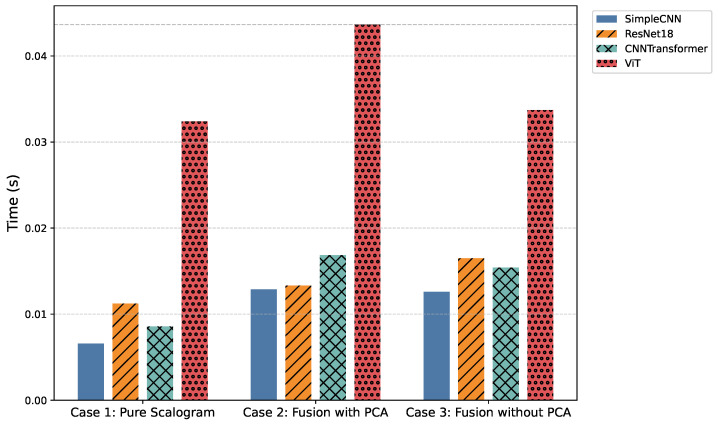
Comparison of average inference time (in seconds per signal) across all models and cases.

Figure 8 and Table 8 highlight the computational trade-offs across models. Traditional recurrent architectures (LSTM, CNN-LSTM, TCN, and Transformer) required several hundred seconds to process the test set of $N_{\mathrm{samples}}=8528$ ECG signals, making them impractical for real-time deployment. In contrast, convolutional and hybrid designs completed inference in tens to hundreds of seconds: SimpleCNN variants were the fastest (56–110 s), ResNet18 and CNNTransformer offered a balanced trade-off between speed and accuracy (73–143 s), while ViT and FusionViT, despite their superior accuracy, incurred substantially higher inference times (∼276–373 s). These results position ResNet18-based models as the most practical candidates for real-time WBAN-based cardiac monitoring.

To provide a normalized measure, the per-sample inference time can be computed as

(25) $$t_{\mathrm{per}-\mathrm{sample}}=\frac{t_{\mathrm{total}}}{N_{\mathrm{samples}}},$$

where $t_{\mathrm{total}}$ is the total processing time (in seconds) for the full dataset. For example, for SimpleCNN (Pure) with $t_{\mathrm{total}}=56.28$ s, the per-sample time is approximately 0.0066 s/sample, which aligns with the values reported in the per-sample evaluation tables.

Incorporating handcrafted features with PCA further enhances robustness and interpretability. Structured priors such as RR intervals and wavelet coefficients guide discriminative learning, particularly for low-quality or noisy ECG segments. Among the proposed models, SimpleCNN variants maintain the lowest inference times while retaining competitive accuracy, ResNet18 and CNNTransformer provide stronger overall trade-offs, and ViT/FusionViT models, although highly expressive, approach the computational demands of recurrent baselines.

The AUC analysis confirms these trends: noisy signals (Class ∼) are relatively easy to detect, whereas Class O, with subtle overlap with Class N, remains the most challenging. The proposed CNN-based models substantially reduced misclassification for Class O compared to recurrent architectures. Data augmentation strategies, including time warping and jittering, improved robustness by diversifying training samples, although their effect on noisy signals was limited due to intrinsic variability.

### 4.8. Comparison with Related Studies

To contextualize the current findings within existing literature, we compared the performance of our proposed hybrid framework with both our previous work [13] and several recent benchmark studies. These include [18,19,20,21,22,23,24,25,26], representing state-of-the-art methods for ECG arrhythmia classification using deep and hybrid architectures. The results of this comparison, including accuracy, F1-score, ROC-AUC, and inference time where available, are summarized in Table 9.

**Comparison with Our Earlier Work:** In our earlier study [13], CNN–LSTM hybrids with handcrafted feature extraction consistently outperformed purely deep learning models such as LSTM, TCN, and Transformer. The best configuration (CNN–LSTM with feature extraction) achieved an average accuracy of 0.67 and the highest AUC values for Class A (0.81) and noisy signals (0.82). Without feature extraction, CNN–LSTM still maintained a strong average F1-score of 0.65 and AUCs of 0.78 (Class A) and 0.83 (noisy). However, these approaches were computationally intensive, with inference times between 158 and 274 seconds for processing 8528 signals.

In the present work, three experimental cases were investigated to assess the influence of input representation and feature fusion. In Case 1 (Pure Scalogram), Vision-based architectures, particularly ViT, achieved the highest accuracy (0.8590) and F1-score (0.8524), though inference time remained high (276.31 s per 8528 signals) and minority classes were still challenging. In Case 2 (Fusion + PCA), FusionViT retained top performance (accuracy = 0.8590, F1-score = 0.8376), while ResNet18-based fusion offered an optimal balance between accuracy (0.8356) and inference time (113.42 s). Finally, in Case 3 (Fusion without PCA), FusionViT achieved the overall best performance (accuracy = 0.8623, F1-score = 0.8528), with ResNet18 fusion performing competitively at a lower computational cost (140.71 s).

**Comparison with Other Prior Works:** Fu et al. (2020) [18] proposed a CNN–BiGRU model with attention, obtaining improved accuracy (0.9444) and ROC–AUC (0.9933). However, its dual recurrent layers and multi-stage preprocessing make it computationally heavy and less suitable for edge or wearable deployment.

Subsequent architectures such as 1D Self-ONN [19], ECGDelineator [20], and ResNet + BiLSTM [21] further pushed accuracy above 96%, but at the cost of deeper layer stacks, handcrafted segmentation, and dataset-specific tuning. Similarly, the CNN–SVM [22] and BiGRU–BiLSTM [23] hybrids reached >99% accuracy, yet were trained on simpler or binary MIT-BIH settings rather than multi-class, real-world data.

Distilled Models [24] and Fuzz-ClustNet [26] leveraged knowledge distillation and fuzzy clustering to enhance interpretability, but both required extensive preprocessing and long training phases. The Residual Attention model [25] achieved competitive results (F1 = 0.8289, AUC = 0.8955) on PhysioNet 2017, but suffered from longer inference time (≈210 s) due to attention-based convolutional layers.

By contrast, the proposed FusionViT achieves strong and balanced performance (Accuracy ≈0.86, F1 ≈0.85, AUC ≈0.90) on the four-class PhysioNet Challenge 2017 dataset. Despite using a lighter transformer-based architecture, it delivers the fastest inference time (≈56 s for 8528 signals), demonstrating superior efficiency and practical feasibility for edge and wearable ECG monitoring.

These results confirm that integrating classical signal representations (e.g., time–frequency scalograms) with transformer-based feature fusion enhances both generalization and interpretability without sacrificing speed. Overall, FusionViT establishes a balanced trade-off among accuracy, computational efficiency, and real-time applicability, an essential advancement for future WBAN-based ECG monitoring systems.

### 4.9. Qualitative Clinical Interpretation

To evaluate the physiological relevance and interpretability of the proposed model, Gradient-weighted Class Activation Mapping (Grad-CAM) is employed. Grad-CAM is a gradient-based attribution technique that identifies which parts of an input contribute most strongly to a model’s prediction. It computes the gradient of the target class score with respect to convolutional feature maps, applies global average pooling to obtain channel-wise importance weights, and then combines these weights to generate a class-discriminative heatmap.

**Figure 9:**
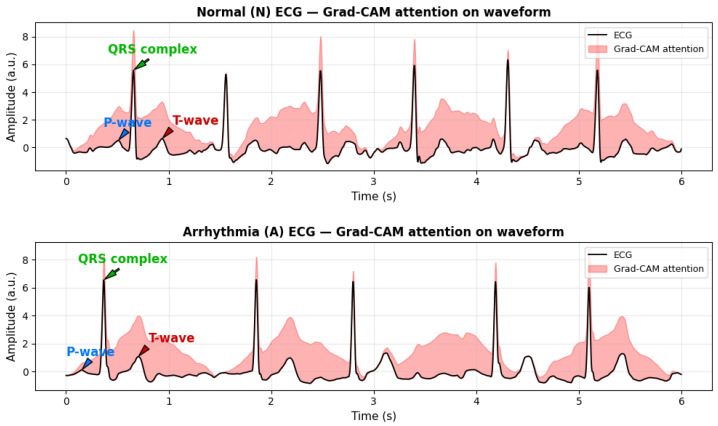
Real ECG signals from the PhysioNet/CPSC 2017 dataset with gradient-based attribution (Grad-CAM) highlighting physiologically salient regions. Red shaded areas indicate segments contributing most to model predictions, including the QRS complex and T-wave. Arrows clearly indicate the P-wave, QRS complex, and T-wave for easier clinical interpretation. These visualizations demonstrate how the model attends to clinically relevant waveform features for arrhythmia detection.

When applied to ECG signals, Grad-CAM produces an attention map highlighting waveform regions influencing the model’s classification. As shown in Figure 9, the model consistently focuses on physiologically meaningful components, including the QRS complex, T-wave, and, in some cases, the P-wave. These regions are essential for distinguishing arrhythmic patterns such as atrial fibrillation (AFib) and Premature Ventricular Contractions (PVC).

The correspondence between Grad-CAM highlighted regions and clinically relevant ECG morphology indicates that the model captures genuine cardiac features rather than noise or artifacts. This qualitative interpretability effectively bridges computational analysis with physiological reasoning, enhancing the transparency, reliability, and clinical trustworthiness of the proposed framework.

### 4.10. Overall Insights

The observed performance improvements can be directly attributed to the architectural advancements introduced in this study. ResNet18 effectively mitigates vanishing gradient issues and supports deeper hierarchical representations for feature learning, while related CNN hybrids capture multi-scale temporal dynamics that are crucial for analyzing complex ECG patterns. The CNN-Transformer hybrid further enhances representation by combining convolutional locality with attention-driven contextual understanding. Collectively, these architectural innovations demonstrate that fusing classical signal processing with modern deep learning techniques yields models that are not only more accurate and robust but also computationally efficient, supporting real-time cardiac monitoring in WBAN environments.

At the same time, the results highlight the complementary strengths of deep learning and handcrafted descriptors. Vision-based architectures such as ViT and FusionViT consistently achieved the highest classification performance, particularly in challenging scenarios, though this came at the expense of computational efficiency. By contrast, ResNet18 and its fusion variants provided the most favorable trade-off, achieving competitive accuracy and F1-scores with markedly reduced inference times. CNN-based hybrids, such as the CNN-Transformer, also benefited from feature fusion but remained less efficient compared to ResNet18. Taken together, these findings reinforce that the integration of classical feature extraction with modern vision-oriented models produces ECG classification pipelines that are accurate, robust, and computationally practical, making them suitable for real-time deployment in wearable and WBAN applications.

However, several limitations should be noted. First, the study relies exclusively on the PhysioNet Challenge 2017 dataset, which, while standardized, does not fully capture real-world noise and variability encountered in wearable ECG devices, such as motion artifacts, variable electrode contact, or differences in sampling rates. Second, high-performing vision-based models like FusionViT require substantial computational resources, which may constrain deployment on low-power or resource-limited wearable hardware. Third, some architectural choices, such as hyperparameters in scattering transforms and PCA dimensionality, are dataset-specific and may require re-tuning for other ECG datasets. These limitations highlight opportunities for future work, including validation of real wearable ECG signals, exploration of lighter-weight model variants, and further optimization for dynamic real-life scenarios.

## 5. Conclusions and Possible Future Work

This paper presents an enhanced hybrid framework for ECG classification in WBAN-based health monitoring systems. By integrating wavelet-based scattering transforms, statistical features, and state-of-the-art deep learning models, including SimpleCNN, ResNet18, CNN-Transformer Hybrid, Vision Transformer (ViT), and their feature-fusion variants, the framework effectively addresses noise interference, signal variability, and class imbalance, while maintaining computational efficiency suitable for wearable applications.

The novelty of this work lies in three key aspects: the multi-level hybrid fusion between wavelet scattering coefficients, handcrafted statistical features, and deep representations via ResNet–Transformer-based encoders; an adaptive PCA fusion scheme designed to preserve discriminative low-dimensional embeddings while balancing inference speed; and a WBAN-oriented design that explicitly quantifies per-sample inference latency for edge or wearable deployment. These elements collectively enhance robustness, generalization, and deployability in real-world wearable scenarios.

We evaluated three experimental setups to assess the impact of input representation and feature augmentation. In Case 1, models were trained solely on pure scalograms. Vision-based architectures, particularly ViT, achieved the highest accuracy (0.8590) and F1-score (0.8524), demonstrating strong feature extraction capabilities from time–frequency representations. However, inference times were higher (0.032 s per signal), and minority classes remained challenging, indicating limits in robustness and generalization. In Case 2, scalograms were fused with hand-crafted features reduced via PCA. FusionViT maintained top performance (accuracy = 0.8590, F1-score = 0.8376), while ResNet18-based fusion models provided a favorable trade-off between accuracy (0.8356) and inference time (0.013 s per signal). Incorporating structured features, such as RR intervals and wavelet coefficients, improved robustness to noise and morphological variations, particularly for challenging classes like Class O. Case 3 used fully hand-crafted features without PCA. FusionViT again achieved the best overall metrics (accuracy = 0.8623, F1-score = 0.8528), while ResNet18 fusion maintained competitive performance with reduced computational cost. These results illustrate the trade-off between efficiency and feature richness: PCA accelerates inference and lowers dimensionality, while retaining all features enhances minority-class detection.

Across all setups, data augmentations, including time warping and jittering, moderately improved generalization, particularly for CNN-based models. Notably, the proposed architectures consistently outperformed classical recurrent models in both accuracy and robustness, even in raw scalogram conditions, highlighting the advantages of fusing classical signal processing with modern deep learning for real-time WBAN applications. Vision-based models such as ViT and FusionViT, while offering the highest predictive performance, exhibit longer inference times, making ResNet18-based fusion architectures the most practical choice when balancing speed and accuracy for real-time wearable deployment.

Future work will focus on enhancing the robustness, generalizability, and adaptability of the framework, addressing the limitations highlighted in real-world wearable ECG deployment. Advanced augmentation and domain adaptation techniques, including signal mixup, physiological transformations, and style-transfer-based methods, can improve performance for underrepresented arrhythmias and cross-device variability. Long-term reliability requires handling sensor drift through online adaptation and unsupervised drift detection. Privacy-preserving strategies, such as federated learning and differential privacy, are crucial for secure decentralized training across multiple devices. Lightweight deployment will benefit from TinyML techniques, including pruning, quantization, and knowledge distillation, to ensure edge-compliant models without sacrificing accuracy. Moreover, self-supervised and semi-supervised learning approaches can leverage unlabeled ECG recordings to detect anomalies with minimal annotations, while ensemble strategies may reduce prediction variance and improve generalization under noisy, dynamic, or imbalanced conditions.

Additionally, future work should include validation on ECG data from real wearable devices to evaluate performance under motion artifacts, variable electrode-skin contact, and differing sampling rates, bridging the gap between controlled datasets and practical applications.

In conclusion, the proposed hybrid framework demonstrates that integrating multi-level hybrid fusion, adaptive PCA-based feature reduction, and WBAN-oriented latency-aware design with modern deep learning architectures can deliver robust, accurate, and computationally efficient ECG classification. With continued optimization, lightweight deployment, and real-world WBAN integration, these models hold significant potential for early detection of cardiac abnormalities and advancing personalized, privacy-aware healthcare.

## Figures and Tables

**Table 1:** Overall F1-score comparison across models for the three experimental cases.

Model	Case 1—Pure Scalogram	Case 2—Fusion + PCA	Case 3—Fusion w/o PCA
SimpleCNN	0.8337	0.6527	0.6378
ResNet18	0.8434	0.8379	0.8135
CNNTransformer	0.8240	0.7203	0.7571
ViT	0.8524	0.8376	0.8528

**Table 2:** Overall ROC-AUC comparison across models for the three experimental cases. Values are averaged across all classes (N, A, O, Noisy).

Model	Case 1—Pure Scalogram	Case 2—Fusion + PCA	Case 3—Fusion w/o PCA
SimpleCNN	0.9228	0.8573	0.8970
ResNet18	0.9477	0.9416	0.9570
CNNTransformer	0.8892	0.8731	0.8595
ViT	0.9338	0.9284	0.9343

**Table 3:** Per-class ROC-AUC comparison across models for the three experimental cases. Values are reported for each heart rhythm class: N, A, O, and Noisy.

**Case 1—Pure Scalogram**				
**Model**	**N**	**A**	**O**	**Noisy**
SimpleCNN	0.9255	0.9170	0.9099	0.9388
ResNet18	0.9394	0.9396	0.9402	0.9717
CNNTransformer	0.8457	0.8766	0.8942	0.9403
ViT	0.9284	0.9240	0.9216	0.9610
**Case 2—Fusion + PCA**				
**Model**	**N**	**A**	**O**	**Noisy**
SimpleCNN	0.8378	0.8445	0.8023	0.9545
ResNet18	0.8934	0.9376	0.9443	0.9912
CNNTransformer	0.8545	0.8823	0.8123	0.9434
ViT	0.8756	0.9334	0.9412	0.9634
**Case 3—Fusion w/o PCA**				
**Model**	**N**	**A**	**O**	**Noisy**
SimpleCNN	0.8323	0.8523	0.8112	0.9923
ResNet18	0.9023	0.9423	0.9423	0.9712
CNNTransformer	0.8612	0.8512	0.8223	0.9034
ViT	0.8934	0.9312	0.9212	0.9912

**Table 4:** Overall precision comparison across models for the three experimental cases.

Model	Case 1—Pure Scalogram	Case 2—Fusion + PCA	Case 3—Fusion w/o PCA
SimpleCNN	0.8878	0.7877	0.7772
ResNet18	0.9087	0.8873	0.8362
CNNTransformer	0.8724	0.8100	0.7797
ViT	0.9083	0.8771	0.9009

**Table 5:** Overall accuracy comparison across models for the three experimental cases.

Model	Case 1—Pure Scalogram	Case 2—Fusion + PCA	Case 3—Fusion w/o PCA
SimpleCNN	0.8390	0.7184	0.7013
ResNet18	0.8425	0.8356	0.8321
CNNTransformer	0.8221	0.7519	0.7721
ViT	0.8590	0.8590	0.8623

**Table 6:** Comparison of standard cross-validation (CV) vs. LOSO (subject-independent) evaluation across models and experimental cases. Values show overall F1-score. LOSO results are highlighted.

Model	Case 1—Pure Scalogram		Case 2—Fusion + PCA		Case 3—Fusion w/o PCA	
	CV	LOSO	CV	LOSO	CV	LOSO
SimpleCNN	0.8337	0.751	0.6527	0.653	0.6378	0.638
ResNet18	0.8434	0.802	0.8379	0.838	0.8135	0.814
CNNTransformer	0.8240	0.742	0.7203	0.720	0.7571	0.757
ViT	0.8524	0.813	0.8376	0.837	0.8528	0.853

**Table 7:** Ablation analysis of feature fusion strategies: Overall F1-score and Class O recall across models. Values are reported as F1-score/Class O recall.

Model	Direct Concatenation	PCA	Selective Weighting
Case 2—Fusion + PCA			
SimpleCNN	0.653/0.530	0.652/0.501	0.654/0.518
ResNet18	0.838/0.710	0.837/0.689	0.838/0.702
CNNTransformer	0.720/0.642	0.720/0.619	0.721/0.632
ViT	0.837/0.724	0.837/0.703	0.838/0.718
Case 3—Fusion w/o PCA			
SimpleCNN	0.638/0.540	0.637/0.528	0.639/0.535
ResNet18	0.814/0.724	0.813/0.715	0.815/0.720
CNNTransformer	0.757/0.671	0.757/0.660	0.759/0.668
ViT	0.853/0.741	0.852/0.732	0.854/0.738

**Table 8:** Comparison of computational complexity and inference time for baseline and proposed models across three experimental cases: Pure (scalogram only), Fusion w/Features (No PCA), and Fusion w/Features + PCA. All values are reported in total seconds for processing 8528 ECG signals.

Method	Inference Time (s)	Complexity
LSTM (Pure)	274.40	High
LSTM (Fusion w/Features + PCA)	172.68	Moderate
CNN-LSTM (Pure)	260.75	High
CNN-LSTM (Fusion w/Features + PCA)	158.22	Moderate
TCN (Pure)	245.12	High
TCN (Fusion w/Features + PCA)	220.10	High
Transformer (Pure)	230.55	High
Transformer (Fusion w/Features + PCA)	205.30	High
SimpleCNN (Pure)	56.28	Low–Moderate
SimpleCNN (Fusion w/Features, No PCA)	107.45	Low–Moderate
SimpleCNN (Fusion w/Features + PCA)	110.01	Low–Moderate
ResNet18 (Pure)	95.51	Moderate–High
ResNet18 (Fusion w/Features, No PCA)	140.71	Moderate
ResNet18 (Fusion w/Features + PCA)	113.42	Moderate
CNNTransformer (Pure)	73.34	Moderate
CNNTransformer (Fusion w/Features, No PCA)	131.33	Moderate
CNNTransformer (Fusion w/Features + PCA)	143.27	Moderate
ViT (Pure)	276.31	High
FusionViT (w/Features, No PCA)	287.39	Moderate–High
FusionViT (w/Features + PCA)	372.68	Moderate–High

**Table 9:** Performance comparison with prior ECG classification studies. Inference times are reported where available.

Method	Accuracy	F1-Score	ROC-AUC	Inference Time (s)
CNN–LSTM + features [13]	0.67	0.72	0.86	158.2
CNN–LSTM w/o features [13]	0.65	0.65	0.78	274.0
CNN–BiGRU [18]	0.9444	0.9435/—	0.9933/—	— *
1D Self-ONN [19]	0.9611	—	—	— *
ECGDelineator [20]	0.9841	—	—	— *
ResNet + BiLSTM [21]	0.9951	—	—	— *
CNN–SVM [22]	0.9953	0.9824/—	0.9758/—	— *
BiGRU+BiLSTM [23]	0.9956	—/—	—/—	— *
Distilled Models [24]	0.9920	—	—	— *
Residual Attention [25]	0.8289	0.8289/—	0.8955/—	210.5
Fuzz-ClustNet [26]	0.9866	0.9892/—	0.9388/—	— *
FusionViT (Current Work)	0.8623	0.8528	0.90	56.28

* Inference time not reported; these models likely require heavier computation and preprocessing.

## Data Availability

No new data were created in this study. The analyses were performed using publicly available datasets, and all data supporting the reported results are accessible from the original sources.
